# Comparing tacrolimus level monitoring in peripheral blood mononuclear cells and whole blood within one year after kidney transplantation: a single-center, prospective, observational study

**DOI:** 10.3389/fphar.2025.1622702

**Published:** 2025-06-11

**Authors:** Jia You, Rui Chen, Yuhui Chai, Xue Wang, Wenmin Xie, Yunyun Yang, Kaile Zheng, Lizhi Chen, Zhuo Wang, Xuebin Wang

**Affiliations:** ^1^ Department of Pharmacy, Shanghai Children’s Hospital, School of Medicine, Shanghai Jiao Tong University, Shanghai, China; ^2^ Department of Pharmacy, Shanghai Changhai Hospital, Naval Medical University, Shangha, China; ^3^ School of Pharmacy, Bengbu Medical University, Bengbu, Anhui, China; ^4^ Department of Kidney Transplantation, Children’s Hospital of Fudan University, Shanghai, China; ^5^ Department of Organ Transplantation, Shanghai Changhai Hospital, Naval Medical University, Shanghai, China; ^6^ Shanghai Altimetria Information Technology LLC, Shanghai, China

**Keywords:** kidney transplantation, tacrolimus, intra-patient variability, peripheral blood mononuclear cell, allograft function, de novo donor-specific antibody

## Abstract

**Background:**

Tacrolimus, a key immunosuppressant for kidney transplant recipients, is traditionally monitored through whole-blood trough concentrations. However, this approach may not accurately reflect lymphocyte tacrolimus levels, limiting its predictive value for allograft function and rejection. Monitoring tacrolimus levels in peripheral blood mononuclear cells (PBMCs) offers a potentially more precise alternative, though its clinical value remains unclear. This study aimed to compare tacrolimus intra-patient variablity (IPV), allograft function, and *de novo* donor-specific anti-HLA antibody (dnDSA) status between PBMC-based and whole-blood tacrolimus monitoring methods to assess whether PBMC monitoring provides greater clinical utility.

**Methods:**

This single-center, prospective, observational, non-interventional study enrolled kidney transplant recipients between November 2021 and February 2023. At six follow-up time points after transplantation (Day 7, Day 14, Month 1, Month 3, Month 6, and Month 12), tacrolimus levels in PBMCs and whole blood were measured, and IPVs in both matrices were calculated. Pearson’s or Spearman’s correlation analyses were used to evaluate (1) the relationship between tacrolimus levels in PBMCs and whole blood, (2) their association with allograft function, and (3) the correlation of tacrolimus IPV with allograft function and dnDSA status.

**Results:**

A total of 60 kidney transplant recipients were included. Within 1-year post-transplantation, the PBMC tacrolimus levels were 3.6% of whole-blood levels (P < 0.01). Tacrolimus levels in PBMCs and whole blood showed positive correlations across six-time points, with statistically significant correlations on Day 7, Day 14, Month 3, and Month 6 (P < 0.05). Notably, PBMC tacrolimus levels demonstrated stronger associations with creatinine clearance and estimated glomerular filtration rate at multiple timepoints compared to whole-blood measurements. Patients with dnDSA exhibited significantly higher IPV in PBMC tacrolimus levels than dnDSA-negative counterparts (P < 0.05), a pattern not observed in whole-blood analysis.

**Conclusion:**

Monitoring tacrolimus levels and IPVs in PBMCs provides greater insight into allograft function and dnDSA status than whole-blood measurements. These findings suggest that PBMC-based tacrolimus monitoring may enhance clinical value in managing kidney transplant recipients.

## 1 Introduction

Kidney transplant recipients require lifelong immunosuppressive therapy, necessitating a delicate balance between mitigating immunosuppressant toxicity and preventing antibody-mediated rejection (ABMR) (Ochando et al., 2020; Levin et al., 2024). Tacrolimus, often combined with mycophenolic acid and corticosteroids, is prescribed to over 90% of kidney transplant recipients (Brunet et al., 2019). Tacrolimus plays a critical role in preventing and managing rejection by inhibiting T-cell activation through suppression of interleukin-2 (IL-2) expression (Brunet et al., 2019). However, its narrow therapeutic window, coupled with substantial intra- and interindividual pharmacokinetic and pharmacodynamic variability, presents challenges in clinical management. To address this, therapeutic drug monitoring (TDM) is used to maintain tacrolimus trough levels within target ranges, thereby minimizing the risks of over- or underexposure (Brunet et al., 2019).

Whole-blood tacrolimus trough concentrations (C0) are widely utilized as a key indicator to guide individualized dosing (Guo et al., 2024). Despite rigorous TDM, the incidence of acute rejection or tacrolimus toxicity remains at 8%–15% within the first year after kidney transplantation, even when whole-blood levels fall within the therapeutic range (Wallemacq et al., 2009). Tacrolimus exhibits a compartmentalized distribution, with 85% residing in red blood cells, 14% in plasma, and only 1% in lymphocytes (Sallustio, 2021). Consequently, routine measurement of whole-blood C0 may not adequately reflect drug concentrations in target cells, such as lymphocytes, which mediate immunosuppression (Bazin et al., 2010; Brunet et al., 2019). Insufficient intracellular tacrolimus levels can lead to rejection, allograft injury, failure, and even mortality (Guo et al., 2024). Since lymphocytes account for 70%–90% of peripheral blood mononuclear cells (PBMCs), tacrolimus levels in PBMCs can be a surrogate marker for its distribution in lymphocytes. Studies by Capron et al. demonstrated that PBMC tacrolimus levels were significantly associated with rejection severity in liver transplant recipients and could serve as early predictors of transplant success (Capron et al., 2007; Capron et al., 2012). High intra-patient variability (IPV) in tacrolimus exposure, reflected by fluctuations in trough concentrations (CV%), is a critical concern in transplant management. While previous studies have linked whole-blood tacrolimus IPV to impaired allograft function, the clinical relevance of IPV in PBMCs remains unknown.

Preliminary research in kidney and other organ transplant recipients has further suggested that monitoring lymphocyte tacrolimus levels may offer enhanced clinical value (Coste and Lemaitre, 2022). However, prior studies have often been limited by small sample sizes, short follow-up periods, or insufficient examination of intracellular tacrolimus levels’ influence on key clinical outcomes (Ghareeb and Akhlaghi, 2015; Jankowska et al., 2021; Coste et al., 2022; Wang et al., 2022; Collins et al., 2023; Xu et al., 2023). Specifically, the relationship between tacrolimus IPV in PBMCs and outcomes such as allograft function, *de novo* donor-specific antibody (dnDSA) formation, and rejection remains unclear (De Nicolo et al., 2021)^.^

This single-center, prospective, observational, noninterventional study was conducted to investigate whether PBMC-based tacrolimus monitoring provides greater clinical utility for kidney transplant recipients. The objectives were to explore the relationship between PBMC and whole-blood tacrolimus concentrations and to compare allograft function, tacrolimus IPV, dnDSA status, and rejection rates under these two monitoring approaches.

## 2 Methods

### 2.1 Study design and patients

This single-center, prospective, non-interventional, observational clinical study was conducted at Shanghai Changhai Hospital. Eligible participants included patients who underwent allogeneic kidney transplantation between November 2021 and February 2023. The study enrolled kidney transplant recipients treated with a tacrolimus-based immunosuppressive regimen and undergoing regular tacrolimus TDM.

Exclusion criteria included pharmacologic kidney injury, discontinuation of tacrolimus during follow-up, failure to receive a transplant, or multi-organ transplantation. Recipients who died or experienced kidney graft failure during the study period were also excluded.

### 2.2 Ethical approval

The study protocol was approved by the Ethics Committee of Shanghai Changhai Hospital (CHEC 2021-133). Prior to enrollment, written informed consent was obtained from all participants. The study was registered with the China Clinical Trial Registration Center under the WHO International Clinical Trials Registry Platform (ChiCTR20003714).

### 2.3 Evaluation indicators

Clinical outcomes assessed included serum creatinine (Scr), creatinine clearance (Ccr), estimated glomerular filtration rate (eGFR), dnDSA, biopsy-confirmed rejection, and the coefficient of variation (CV%) as a measure of tacrolimus IPV. eGFR was calculated using the Modification of Diet in Renal Disease (MDRD) equation: eGFR (mL/(min*1.73 m^2^) = 186 × (Scr)^−1.154^ × (age)^−0.203^ × (0.742 if female), adapted for the Chinese population.

### 2.4 Immunosuppressive regimen

All participants received a tacrolimus-based immunosuppressive regimen. Induction therapy included either basiliximab or rabbit anti-human thymocyte immunoglobulin. Maintenance therapy comprised tacrolimus, mycophenolate mofetil or mycophenolate sodium enteric-coated tablet, and corticosteroids (e.g., methylprednisolone sodium). Initial tacrolimus dosing was weight-based, with subsequent adjustments guided by TDM and target trough concentrations in whole blood. Target tacrolimus concentrations in whole blood were 10–15 ng/mL during the first-month post-transplant, 8–12 ng/mL within the first 3 months post-transplant, and 5–10 ng/mL from six to 12 months post-transplant.

### 2.5 Blood sampling and tacrolimus measurement

Follow-up assessments were scheduled at 7 ± 2 days, 14 ± 2 days, 1 ± 0.25 months, 3 ± 0.25 months, 6 ± 0.25 months, and 12 ± 0.25 months post-transplantation. Based on clinical considerations, adjustments to these intervals were made as needed.

At each follow-up, two EDTA-K2–anticoagulated peripheral blood samples were collected before the morning tacrolimus dose: 2 mL for whole-blood tacrolimus measurement and 6 mL for PBMC isolation. Whole-blood tacrolimus concentrations were determined using a chemiluminescent microparticle immunoassay (CMIA) with the ARCHITECT i1000SR analyzer (Abbott, United States). PBMCs were isolated via Ficoll gradient centrifugation, and PBMC tacrolimus levels were measured using liquid chromatography-tandem mass spectrometry (LC-MS/MS) (Chen et al., 2020).

### 2.6 Data collection

Collected demographic and clinical data included sex, age, weight, height, primary kidney disease, daily tacrolimus dose, donor source, delayed graft function, human leukocyte antigen (HLA) mismatch, and donor type (living or deceased). Laboratory data such as tacrolimus trough concentrations, complete blood count, liver function, and kidney function were retrieved from the TDM information management system.

### 2.7 Statistical analysis

Statistical analyses were conducted using SPSS software (version 21, IBM, United States). The Shapiro-Wilk test was used to assess the normality of continuous variables. Normally distributed data are expressed as mean ± standard deviation (SD), while non-normally distributed data are presented as median (M) with interquartile range (Q). Categorical variables are summarized as frequencies and percentages (%).

Correlation analyses were performed using GraphPad Prism 8.0. Pearson’s correlation was applied to normally distributed variables, while Spearman’s rank correlation was used for non-normally distributed or ordinal data. Depending on data distribution, between-group comparisons were conducted using either the independent-samples t-test or nonparametric tests. A p-value <0.05 was considered statistically significant. Figures were generated using GraphPad Prism 8.0 or SPSS 21.

## 3 Results

### 3.1 Clinical basic information

A total of 60 kidney transplant recipients were enrolled, with a median age of 22.25 years and a mean body weight of 55.43 kg. Of these, 58 (96.7%) received induction immunotherapy with antilymphocyte globulin and basiliximab before transplantation. During the follow-up period, 14 recipients were tested for dnDSAs, with 7 testing positive. One recipient experienced biopsy-confirmed rejection (Table 1).

**TABLE 1:** Basic characteristics of kidney transplant recipients (n = 60).

Category	Description	Values
Baseline Characteristics	Male	39 (65%)
	Age (years)	22.25 (12, 35.5)
	Weight (kg)	55.43 ± 17.12
Source of Donor	Deceased donor	51 (85%)
	Living donor	9 (15%)
Induction Immunosuppressant	Anti-thymocyte globulin	33 (55%)
	Basiliximab	25 (41.7%)
Maintenance Immunosuppressant	MPA dose (g/day)	0.56 ± 0.28
	GC dose (mg/day)	419.33 ± 110.71
	TAC dose (mg/day/kg)	0.05 (0.04, 0.08)
Tacrolimus Level	Whole blood (ng/mL)	13.98 ± 4.07
	PBMC (10^–7^ ng/mL)	0.51 (0.36, 0.80)
Donor Specific Antibody	dnDSA	11 (18.33%)
Rejection	ABMR and TCMR	1 (1.67%)
Kidney Function	Scr (μmol/L)	118 (89.25, 145.5)
	Ccr (mL/min)	57.41 ± 24.07
	eGFR (ml/min/1.73 m^2^)	63.87 (48.18, 85.3)
	Blood urea nitrogen (mmol/L)	9.5 (7.4, 12.23)
T Lymphocyte Subset	Ratio of CD4+/CD8+	1.22 (0.62, 1.89)
Liver Function	Alanine aminotransferase (U/L)	14 (10, 19)
	Aspartate aminotransferase (U/L)	13 (11, 17)
	Total protein (g/L)	63.08 ± 5.8
	Albumin (g/L)	39.28 ± 3.91
	Total bilirubin (g/L)	6.35 (5.1, 7.98)
	Direct bilirubin (g/L)	2 (1.63, 2.88)
	Total bile acid (μmol/L)	4.9 (2.78, 8.05)
Blood Glucose	Fasting blood glucose (mmol/L)	5.15 (4.7, 6.03)
Blood Lipid	Total cholesterol (mmol/L)	4.33 ± 0.86
	Triglyceride (mmol/L)	1.65 (1.29, 2.26)
	High-density lipoprotein (mmol/L)	1.08 ± 0.24
	Low-density lipoprotein (mmol/L)	2.56 ± 0.76
Blood Cell	Red blood cell (10^12^/L)	3.29 ± 0.51
	Hemoglobin (g/L)	97 (88.25, 107.75)
	Hematocrit (%)	0.31 (0.28, 0.34)
	Platelet (10^9^/L)	244.98 ± 64.54
	White blood cell (10^9^/L)	8.26 (6.38, 10.8)
	Neutrophil (%)	75 (65.6, 82.45)
	Lymphocyte	1 (0.47, 1.9)

^a^ Continuous data are presented as mean ± SD, or median with IQR, depending on variable distribution. Categorical data are presented as n (%).

Abbreviations: ABMR, antibody-mediated rejection; Ccr, creatinine clearance rate; CV, coefficient of variation; dnDSA, *de novo* donor-specific antibody; eGFR, estimated glomerular filtration rate; GC, glucocorticoids; IPV, intra-patient variability; MPA, mycophenolic acid; PBMC, peripheral blood mononuclear cell; Scr, serum creatinine; TAC, tacrolimus; TCMR, T cell-mediated rejection.

**FIGURE 1:**
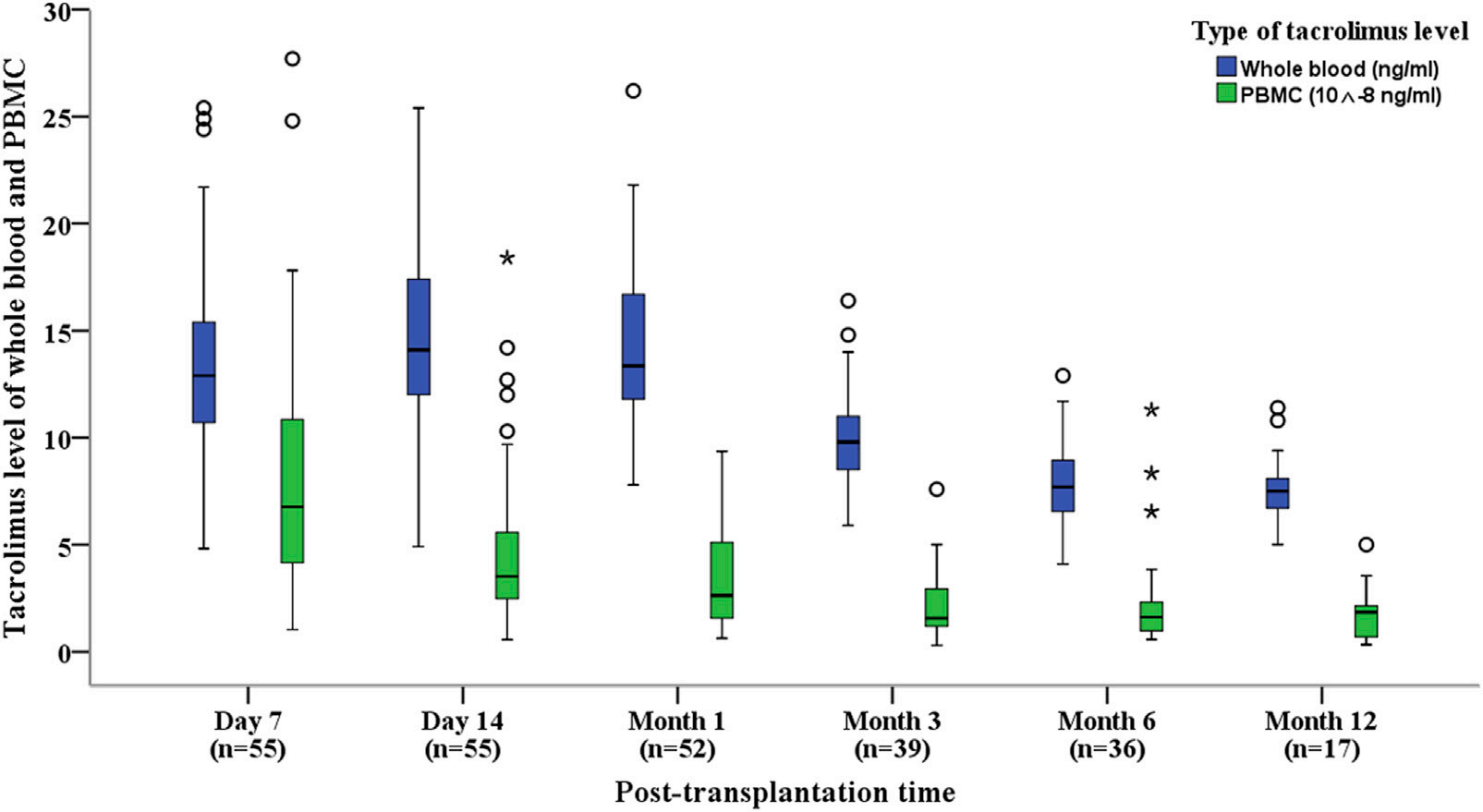
Box plots showing tacrolimus levels in whole blood and PBMCs for kidney transplant recipients at various post-transplantation time points. Box plots display median, interquartile range (IQR).

Tacrolimus concentrations in PBMCs were significantly lower than those in whole blood across the first-year post-transplantation (t = 45.788, P < 0.001; Table 1). Whole-blood tacrolimus concentrations remained relatively stable within the target range of 10–15 ng/mL during Days 7 and 14 and Month one post-transplantation, gradually decreasing to the lower target range of 5–10 ng/mL by Months 3, 6, and 12. By contrast, PBMC tacrolimus concentrations consistently declined throughout the first year (Figure 1).

### 3.2 Changes in tacrolimus concentration, allograft function

Tacrolimus daily doses, concentrations in PBMCs and whole blood, and indicators of allograft function were evaluated at six follow-up time points within the first year after kidney transplantation: Day 7, Day 14, Month 1, Month 3, Month 6, and Month 12 (Table 2).

**TABLE 2:** Data of tacrolimus levels and kidney function within 1 Year for kidney transplant recipients.

Variables	Day 7 (n = 55)	Day 14 (n = 55)	Month 1 (n = 52)	Month 3 (n = 39)	Month 6 (n = 36)	Month 12 (n = 17)
TAC dose (mg/d/kg)	0.09 (0.06, 0.11)	0.07 (0.05, 0.09)	0.05 (0.03, 0.08)	0.04 (0.03, 0.05)	0.03 (0.03, 0.04)	0.03 (0.03, 0.05)
Whole blood TAC level (ng/mL)	12.9 (10.7, 15.45)	13.73 ± 3.83	13.91 ± 3.39	9.73 ± 2.55	8.07 ± 2.25	9.07 ± 2.08
PBMC TAC level (10^–7^ ng/mL)	0.68 (0.44, 1.14)	0.35 (0.25, 0.56)	0.24 (0.16, 0.57)	0.16 (0.12, 0.28)	0.14 (0.09, 0.2)	0.11 (0.05, 0.22)
Scr (μmol/L)	112 (80, 197)	118 (87, 161)	118 (92.75, 149.75)	113.93 ± 32.18	109.89 ± 30.24	100.29 ± 39.5
Ccr (mL/min)	55.07 ± 30.14	58.22 (36.18, 79.3)	54.8 ± 19.87	60.67 ± 19.32	64.4 ± 16.46	63.52 ± 15
eGFR (mL/min/1.73 m^2^)	64.3 (42.32, 94.9)	64.39 (45.2, 89.34)	62.59 (46.4, 81.15)	65.58 ± 22.85	65.36 ± 15.71	70.02 ± 24.59

^a^ Continuous data are presented as mean ± SD, or median with IQR, depending on variable distribution.

Abbreviations: Ccr, creatinine clearance rate; eGFR, estimated glomerular filtration rate; PBMC, peripheral blood mononuclear cell; Scr, serum creatinine; TAC, tacrolimus.

Tacrolimus daily doses progressively decreased at each follow-up interval, mirrored by a corresponding downward trend in PBMC tacrolimus levels. However, whole-blood tacrolimus concentrations showed a noticeable decline at Months 3, 6, and 12 (Figure 1).

Throughout the study period, markers of allograft function (Scr, Ccr, and eGFR) remained stable (Table 2). This stability suggests that despite the divergent trends in tacrolimus levels between PBMCs and whole blood, there was no significant impact on allograft function during the first year post-transplantation.

### 3.3 Correlation between tacrolimus levels in PBMCs and whole blood

**FIGURE 2:**
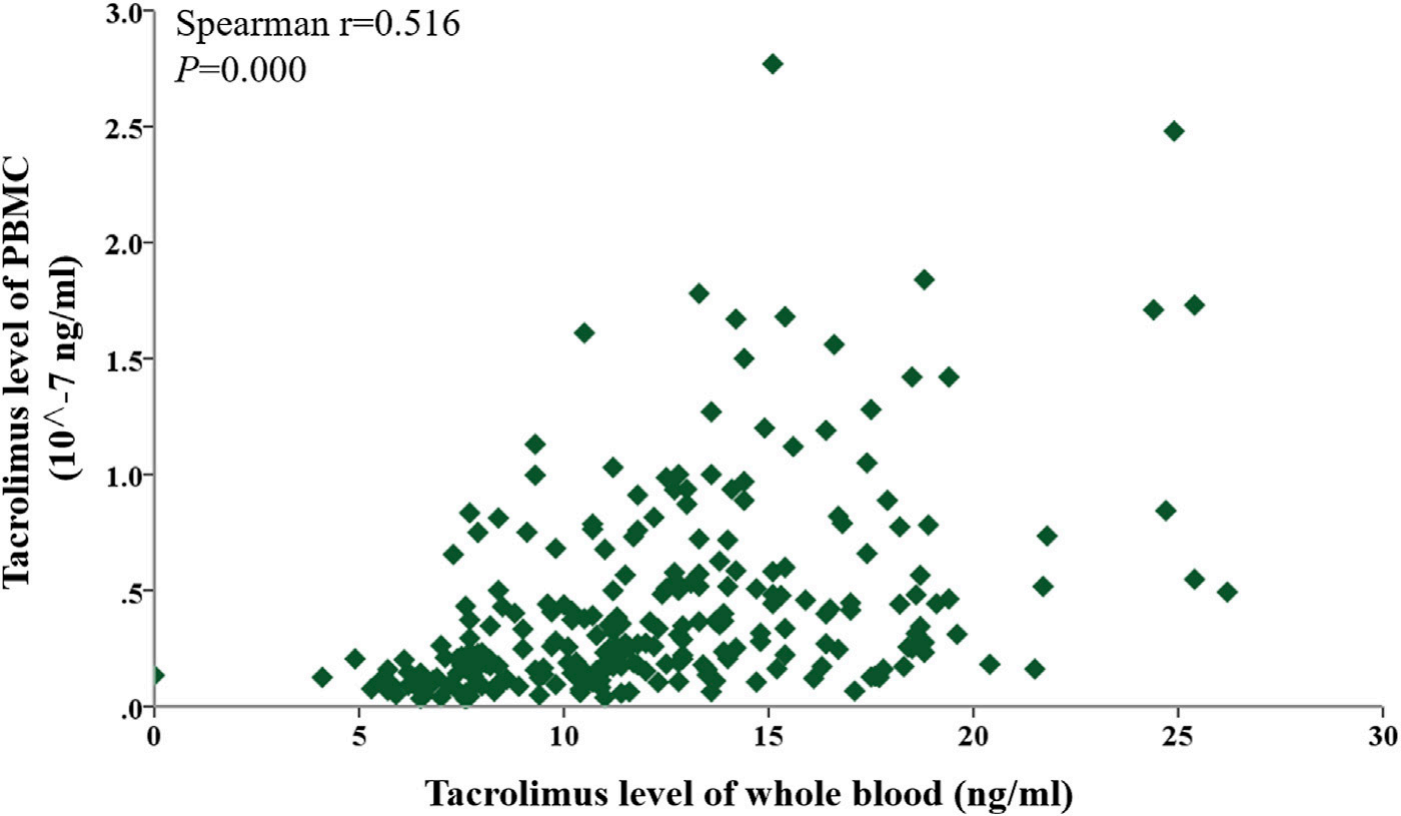
Scatter plot showing the correlation between tacrolimus levels in whole blood and PBMCs within 1 year after kidney transplantation. Correlations were assessed using Spearman’s rank correlation.

Given that tacrolimus levels in PBMCs at the six follow-up time points did not follow a normal distribution (Table 2), a Spearman correlation analysis was conducted to evaluate the relationship between tacrolimus concentrations in whole blood and PBMCs over the first-year post-transplantation. A significant positive correlation was observed overall (P < 0.05), as shown in Figure 2.

**FIGURE 3:**
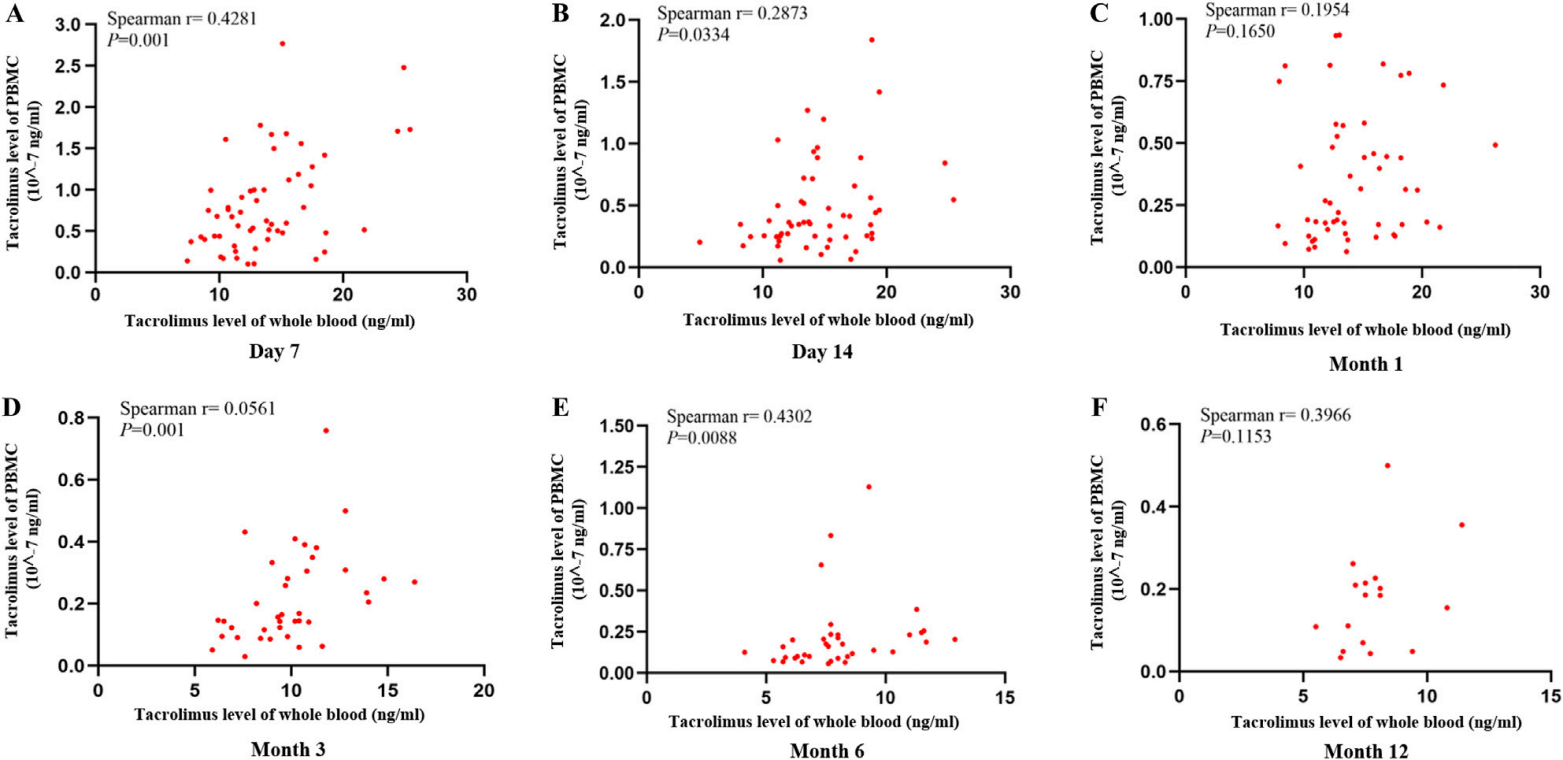
Scatter plot showing the correlation between tacrolimus levels in whole blood and PBMCs at **(A)** Day 7, **(B)** Day 14, **(C)** Month 1, **(D)** Month 3, **(E)** Month 6, **(F)** Month 12 after kidney transplantation. Correlations were assessed using Spearman’s rank correlation.

To assess the associations at each specific time point (Day 7, Day 14, Month 1, Month 3, Month 6, and Month 12), Spearman correlations between whole-blood and PBMC tacrolimus levels were analyzed individually. Significant positive correlations were identified on Day 7, Day 14, Month 3, and Month six post-transplant (Spearman’s r = 0.4281, 0.2873, 0.0561, 0.4302; P < 0.05). Although a positive trend was noted in Months one and 12, these findings did not reach statistical significance (P > 0.05). The correlation coefficients (r = 0.1954–0.4302) were modest, reflecting a weak association between tacrolimus concentrations in PBMCs and whole blood (Figure 3).

### 3.4 Correlations between tacrolimus levels in PBMCs and whole blood and allograft function

Tacrolimus exerts its immunosuppressive effects primarily through T lymphocytes, suggesting that PBMC tacrolimus levels may better reflect transplanted kidney function than whole-blood concentrations. To explore this hypothesis, correlations between tacrolimus levels (in both PBMCs and whole blood) and allograft function indicators (Scr, Ccr, and eGFR) were evaluated. The strength of these correlations was also compared to determine the relative utility of PBMC-based *versus* whole-blood monitoring.

**FIGURE 4:**
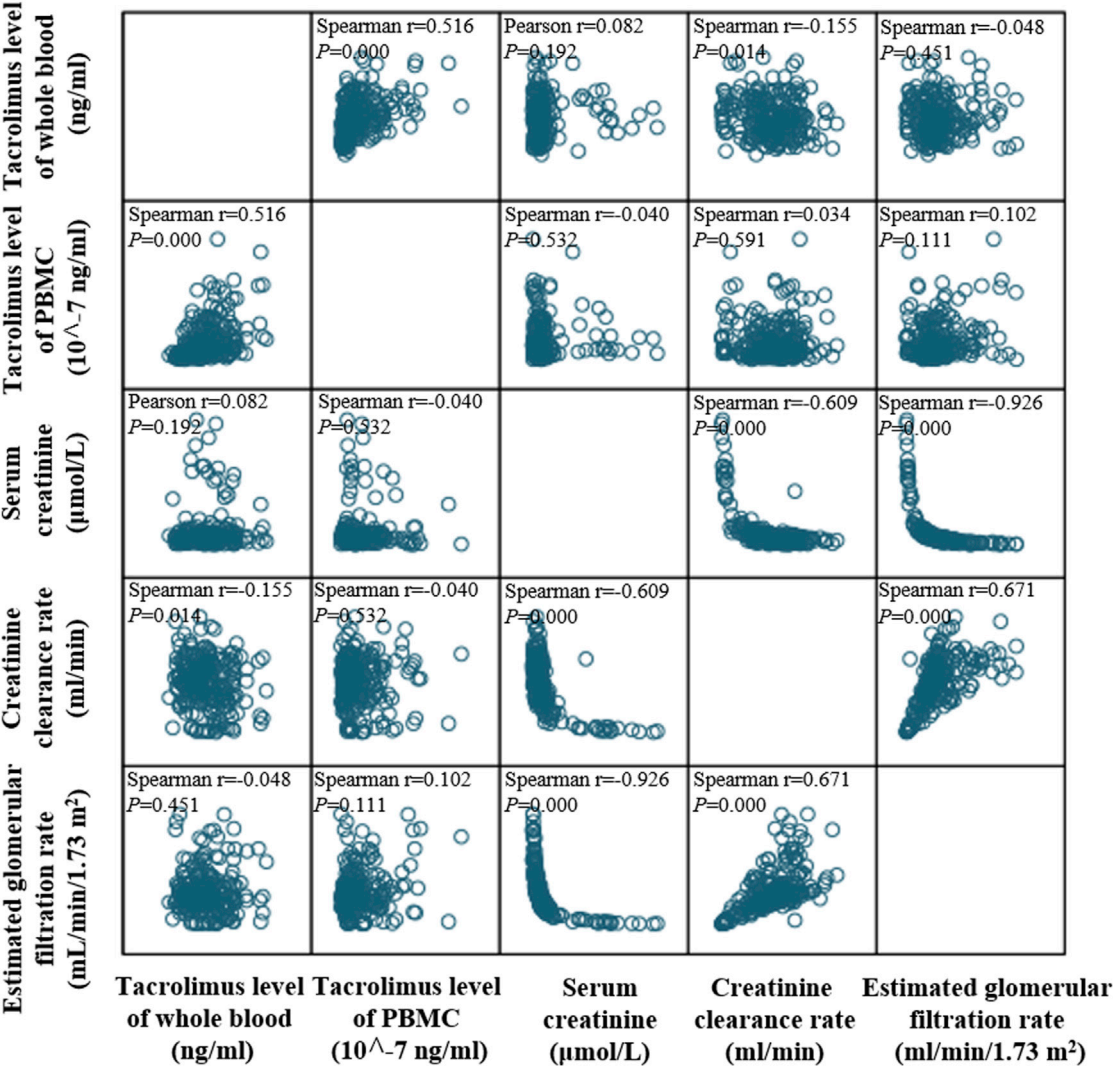
Matrix scatter plot illustrating the distribution and correlation between tacrolimus levels in whole blood and PBMCs within 1 year after kidney transplantation. Correlations were assessed using Spearman’s rank correlation.

PBMC tacrolimus levels demonstrated a negative correlation with Scr (r < 0) and positive correlations with both Ccr and eGFR (r > 0; Figure 4). Additionally, correlations were assessed at six specific time points: Day 7, Day 14, Month 1, Month 3, Month 6, and Month 12 post-transplantation.

#### 3.4.1 Correlations between tacrolimus levels and serum creatinine (Scr)

**FIGURE 5:**
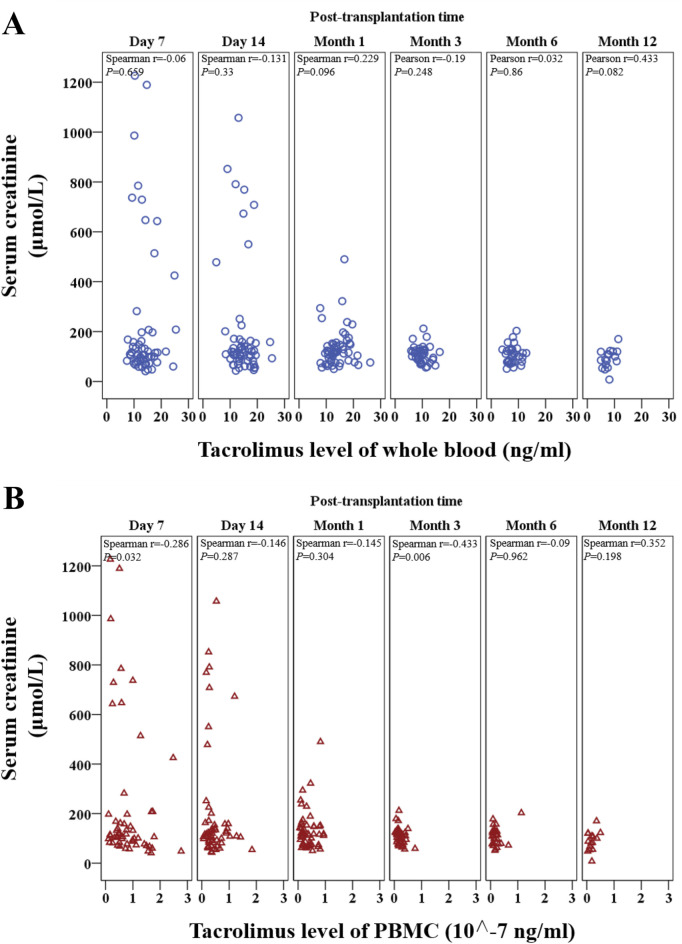
Scatter plot showing the continuous distribution for the correlation between tacrolimus levels and serum creatinine at various time points within 1 year after kidney transplantation. **(A)** Tacrolimus levels in whole blood, **(B)** Tacrolimus levels in PBMCs. Spearman’s correlation analysis was applied at each time point.

Whole-blood tacrolimus levels correlated negatively with Scr on Day 7, Day 14, and Month 3, while positive correlations were observed at Month 1, Month 6, and Month 12. However, none of these correlations reached statistical significance (P > 0.05; Figure 5A).

In contrast, PBMC tacrolimus levels were negatively correlated with Scr at all time points except Month 12. Significant negative correlations were observed on Day 7 and Month 3 (P < 0.05; Figure 5B).

#### 3.4.2 Correlations between tacrolimus levels and creatinine clearance rate (Ccr)

**FIGURE 6:**
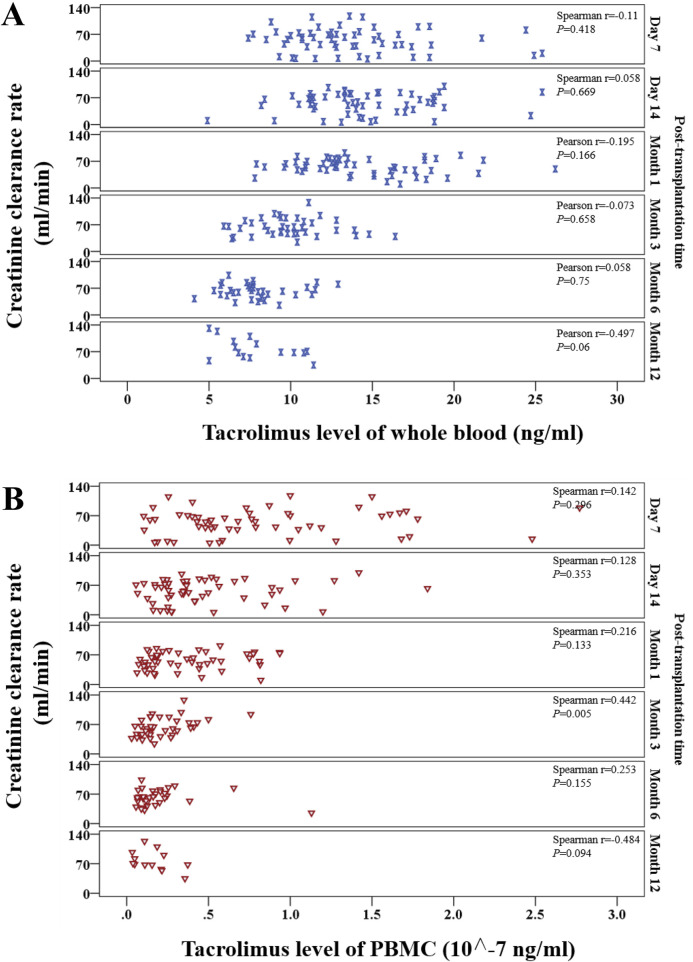
Scatter plot showing the continuous distribution for the correlation between tacrolimus levels and creatinine clearance rate at various time points within 1 year after kidney transplantation. **(A)** Tacrolimus levels in whole blood, **(B)** Tacrolimus levels in PBMCs. Spearman’s correlation analysis was applied at each time point.

Whole-blood tacrolimus levels negatively correlated with Ccr on Day 7, Month 1, Month 3, and Month 12, whereas positive correlations were observed on Day 14 and Month 6. These correlations were insignificant (P > 0.05; Figure 6A).

In contrast, PBMC tacrolimus levels were positively correlated with Ccr at all time points except Month 12. A statistically significant positive correlation was observed at Month 3 (P < 0.05; Figure 6B).

#### 3.4.3 Correlations between tacrolimus levels and estimated glomerular filtration rate (eGFR)

**FIGURE 7:**
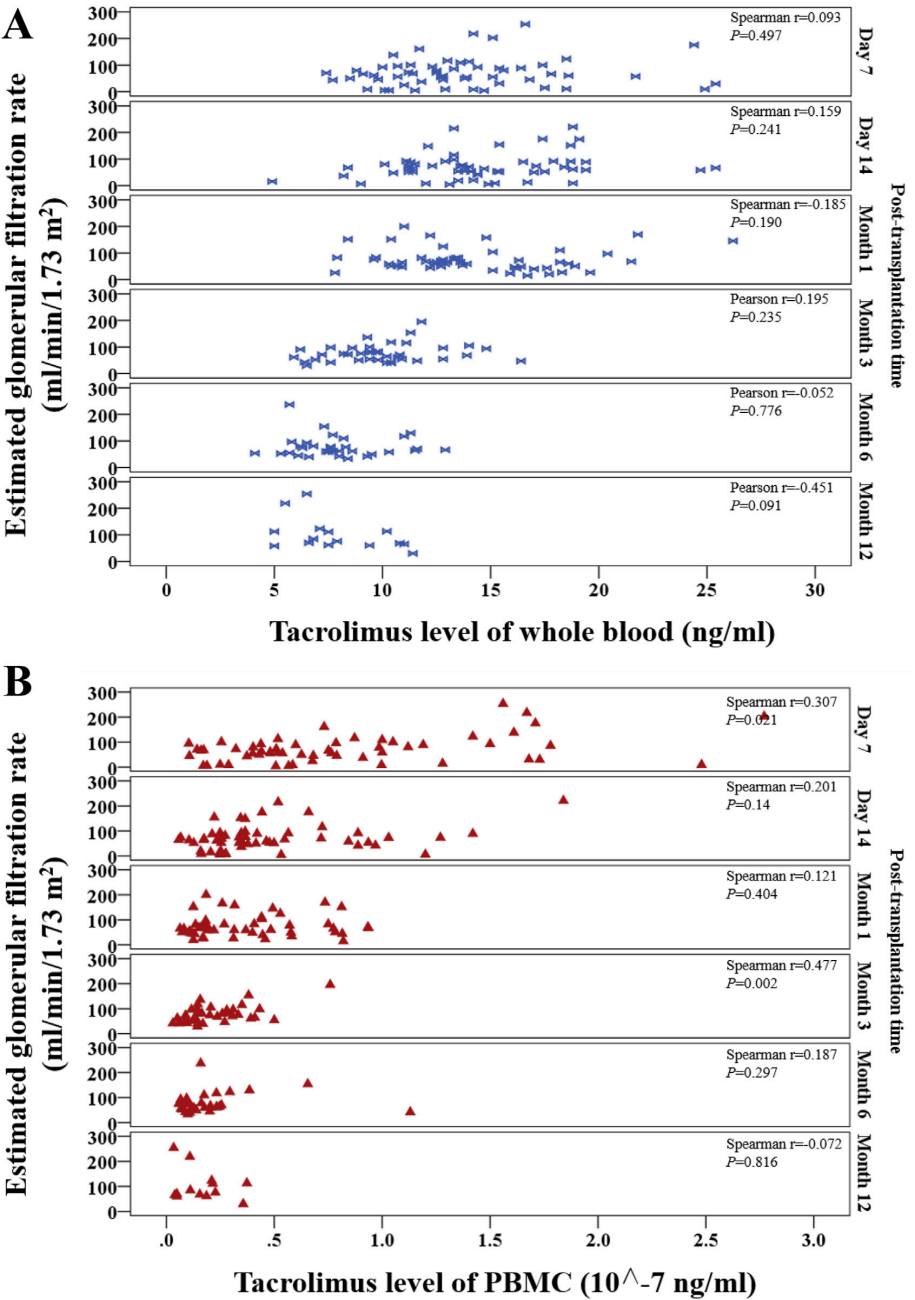
Scatter plot shows the continuous distribution for the correlation between tacrolimus levels and estimated glomerular filtration rate (eGFR) at various time points within 1 year after kidney transplantation. **(A)** Tacrolimus levels in whole blood, **(B)** Tacrolimus levels in PBMCs. Spearman’s correlation analysis was applied at each time point.

Whole-blood tacrolimus levels exhibited positive correlations with eGFR on Day 7, Day 14, and Month 3, while negative correlations were observed at Month 1, Month 6, and Month 12 (Figure 7A).

PBMC tacrolimus levels demonstrated positive correlations with eGFR at all time points except Month 12. Significant positive correlations were observed at Month one and Month 3 (P < 0.05; Figure 7B).

### 3.5 Correlation of tacrolimus IPVs in both PBMCs and whole blood with allograft function

The CV% of tacrolimus IPV in whole blood and PBMCs was calculated at 1-, 3-, 6-, and 12-month post-transplantation to address this. Calculations required at least three drug concentration measurements and complete data for whole-blood and PBMC tacrolimus concentrations, Scr, Ccr, and eGFR.

At 1-, 3-, 6-, and 12-month post-transplantation, the CV% of whole-blood tacrolimus IPV was 23.34% ± 12.45%, 28.51% ± 11.75%, 36.34% ± 11.42%, and 35.16% ± 11.92%, respectively. Corresponding CV% values for PBMC tacrolimus IPV were 48% (29%, 70%), 64.34% (44.48%, 78.26%), 71.96% (58.02%, 89.21%), and 79.08% ± 15.54%, respectively.

Clinical measures of allograft function, including Scr, Ccr, and eGFR, were as follows: 1) Month 1: Scr = 116 (77–148) μmol/L, Ccr = 55.73 ± 19.55 mL/min, eGFR = 65.6 (48.72–82.82) mL/min/1.73 m^2^; 2) Month 3: Scr = 103.14 ± 26.99 μmol/L, Ccr = 67.9 ± 21.56 mL/min, eGFR = 72.43 (54.35–96.08) mL/min/1.73 m^2^; Month 6: Scr = 106.99 ± 41.09 μmol/L, Ccr = 64.23 ± 18.62 mL/min, eGFR = 68.33 (51.81–96.97) mL/min/1.73 m^2^; Month 12: Scr = 84.76 ± 41.66 μmol/L, Ccr = 71.16 ± 27.35 mL/min, eGFR = 118.83 ± 78.7 mL/min/1.73 m^2^.

**FIGURE 8:**
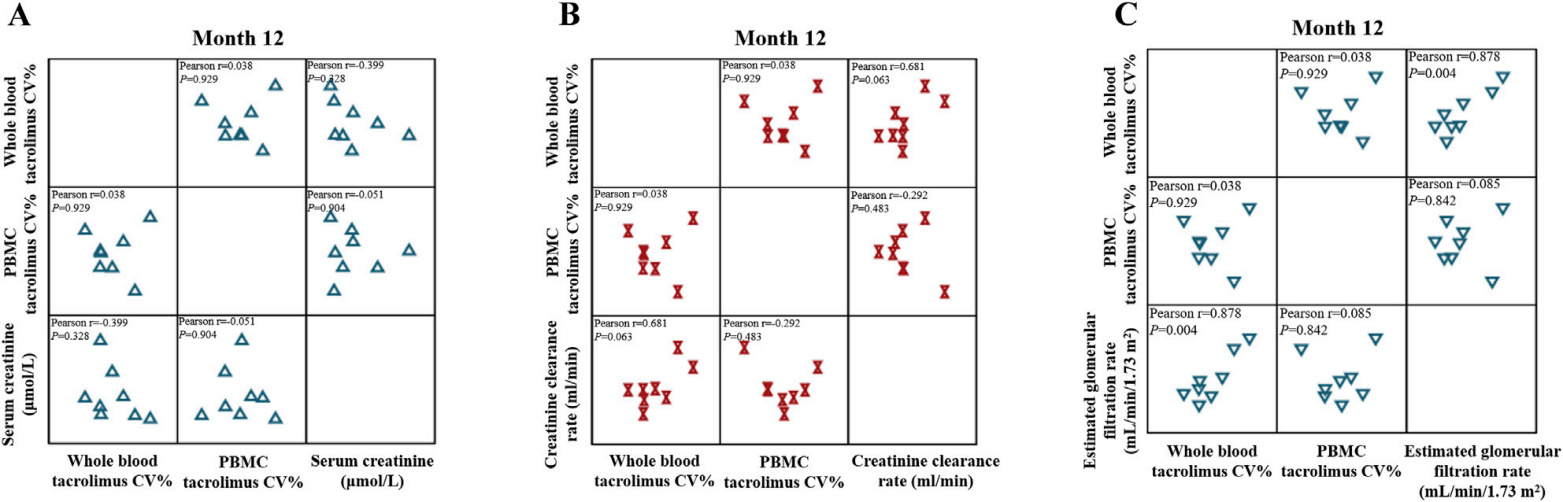
Matrix scatter plot illustrating the correlation between tacrolimus intra-patient variability (IPV) in whole blood and PBMCs with **(A)** serum creatinine, **(B)** creatinine clearance rate, and **(C)** estimated glomerular filtration rate (eGFR) at month 12 after kidney transplantation (n = 8). Pearson’s correlation analysis was applied at each time point.

At Month 12, whole-blood and PBMC tacrolimus IPVs were positively correlated (r > 0). Whole-blood tacrolimus IPV showed a positive correlation with Scr (r > 0), whereas PBMC tacrolimus IPV was negatively correlated with Scr (r < 0; Figure 8A). Similarly, whole-blood tacrolimus IPV was positively correlated with Ccr (r > 0), while PBMC tacrolimus IPV exhibited a negative correlation with Ccr (r < 0; Figure 8B). These results suggest that PBMC tacrolimus IPV adversely affects allograft function in kidney transplant recipients.

eGFR, a more precise indicator of renal function than Ccr, demonstrated positive correlations with whole-blood and PBMC tacrolimus IPVs (r > 0). The correlation coefficient for whole-blood tacrolimus IPV (r = 0.878) was significantly higher than that for PBMC tacrolimus IPV (r = 0.085; Figure 8C).

### 3.6 Correlation of tacrolimus IPVs in both PBMC and whole blood with dnDSA and rejection

#### 3.6.1 Correlation of tacrolimus IPVs in both PBMC and whole blood with dnDSA

**FIGURE 9:**
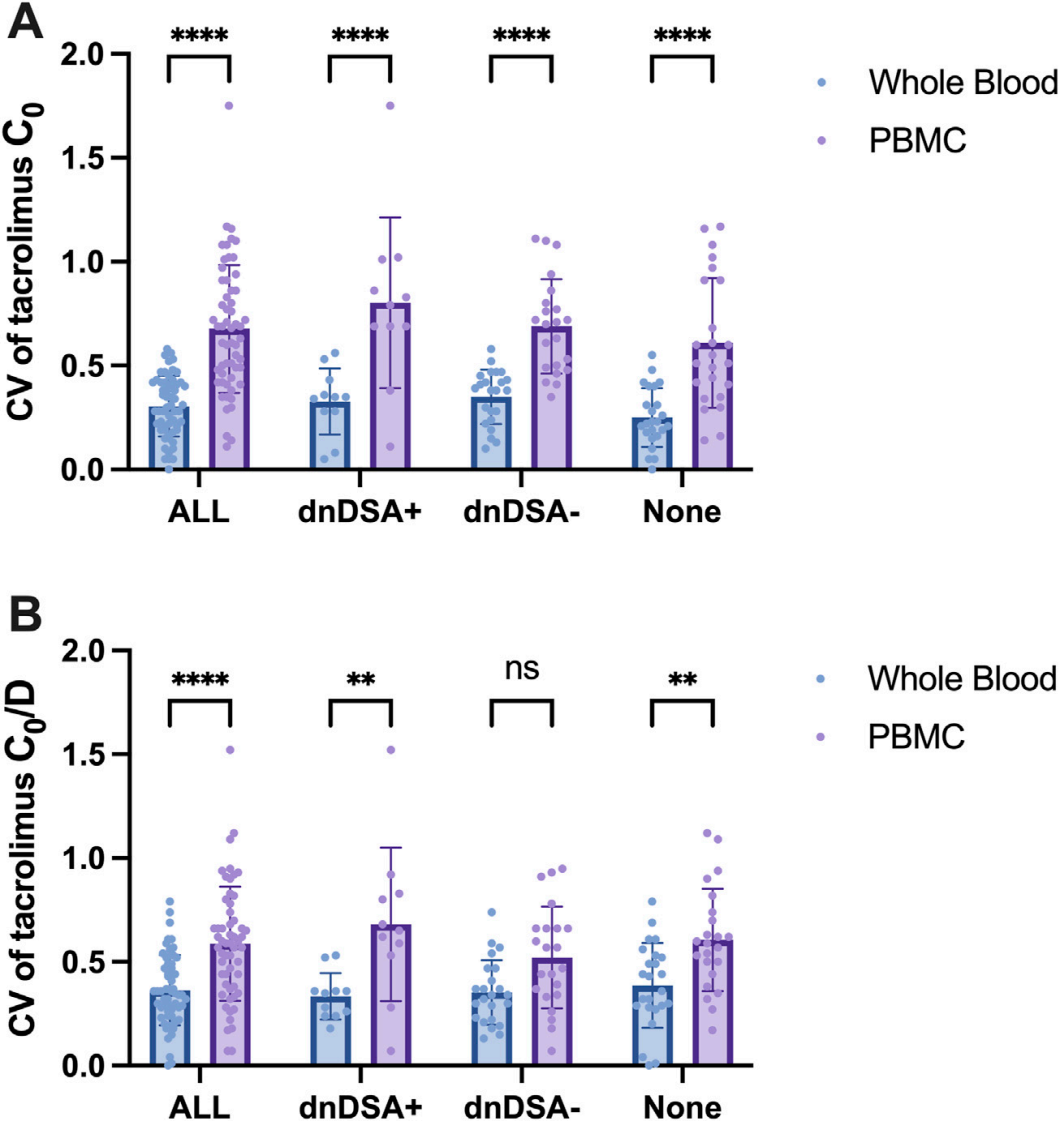
IPV (CV) of Tacrolimus Concentrations in Whole Blood and PBMCs in Kidney Transplant Recipients Stratified by dnDSA Status. **(A)** CV of Tacrolimus C_0_ in Whole Blood and PBMC, **(B)** CV of Tacrolimus C_0_/D in Whole Blood and PBMC. Statistical significance notation across all figures: **p < 0.01, ****p < 0.0001. Two-way ANOVA for multiple comparisons.

A total of 35 kidney transplant recipients underwent dnDSA testing, of whom 11 tested positive. Patients were stratified into four groups: All (all patients), dnDSA+, dnDSA-, and None (patients without dnDSA testing). As illustrated in Figure 9A, the variability in tacrolimus levels within PBMCs was significantly higher than that in whole blood across all groups. However, no statistically significant differences in tacrolimus level variability were observed between the dnDSA+ and dnDSA-groups, either in whole blood (P = 0.9579) or PBMCs (P = 0.3474). After adjusting for potential confounding factors such as daily dose and body weight, which may influence tacrolimus concentrations, it was observed that the variability in tacrolimus levels within PBMCs remained higher than that in whole blood for dnDSA + group. Tacrolimus IPV in dnDSA + group was higher than that in dnDSA-group, but no statistically significant differences (P = 0.1216) (Figure 9B).

#### 3.6.2 Correlation of tacrolimus IPVs in both PBMC and whole blood with rejection

Two kidney transplant recipients were suspected of chronic rejection and subsequently underwent renal biopsy. One recipient was diagnosed with ABMR and T-cell-mediated rejection (TCMR), accompanied by a positive dnDSA status. This recipient exhibited higher tacrolimus IPV in both matrices: whole-blood IPV (40%) surpassed the cohort median (28%), while PBMC IPV (79%) matched the median but fell within the upper quartile (Q3: 89.21%). Despite aligning with the PBMC median, this value reflects high variability relative to the population distribution, categorizing the individual as part of the high-IPV subgroup.

## 4 Discussion

This study represents the first prospective, single-center clinical investigation of kidney transplant recipients with a 1-year follow-up. In addition to exploring the relationship between tacrolimus levels in PBMCs and whole blood, we conducted several novel analyses. These included comparisons of the associations between tacrolimus levels in PBMCs and whole blood with allograft function, the evaluation of tacrolimus IPV in PBMCs *versus* whole blood, and the comparison of the relationships between both IPVs and key clinical outcomes, such as allograft function and dnDSA status. The results suggest that monitoring tacrolimus levels in PBMCs may have greater clinical utility, as PBMC tacrolimus levels demonstrated stronger correlations with allograft function.

In current clinical practice, acute rejection occurs in 10%–15% of kidney transplant recipients despite achieving therapeutic tacrolimus trough concentrations C0 in whole blood (Udomkarnjananun et al., 2023). This observation highlights the limitations of whole-blood tacrolimus levels in accurately predicting rejection (Lemaitre et al., 2020). Conversely, tacrolimus concentrations in PBMCs may be a more sensitive and clinically relevant metric for assessing allograft function. PBMC-based monitoring could improve the prediction of therapeutic efficacy and adverse outcomes (Brunet et al., 2019; Udomkarnjananun et al., 2023; Guo et al., 2024).

However, most existing studies have focused on developing methods for measuring tacrolimus concentrations in PBMCs and examining the correlation between PBMC and whole-blood tacrolimus levels. Few studies have investigated how PBMC-based tacrolimus monitoring might inform precise drug administration and influence clinical outcomes in kidney transplantation (Lemaitre et al., 2020; Udomkarnjananun et al., 2023; Xu et al., 2023). Furthermore, prospective clinical investigations directly comparing the two monitoring modalities and their relationships with clinical outcome measures have been scarce. Our clinical exploratory study addresses this gap by providing evidence supporting PBMC-based tacrolimus monitoring in kidney transplant recipients.

Our study suggests that tacrolimus concentrations in PBMCs offer certain potential advantages. However, the correlation between PBMC and whole-blood tacrolimus concentrations was relatively weak, and the strength of these correlations varied across different time points. The limited sample size and potential confounding factors may have influenced these results. Furthermore, when assessing the relationships between tacrolimus concentration and clinical outcomes such as allograft function, dnDSA, and rejection, PBMC concentrations did not demonstrate significant superiority over whole-blood levels. This may be attributed to the small sample size, the influence of cell membrane transporters (e.g., P-glycoprotein [P-gp]), or other confounding factors such as sample storage conditions (Romano et al., 2018; Coste and Lemaitre, 2022; Udomkarnjananun et al., 2022; Tornatore et al., 2023; Matsumoto et al., 2024; Wang et al., 2024; Yang et al., 2024).

Differences in tacrolimus concentrations within specific cell types of PBMCs (e.g., monocytes, B cells, T cells) compared to whole-blood levels may differentially affect clinical outcomes (Tornatore et al., 2023). Romano et al. (2018) developed an analytical technique using UPLC-MS/MS to measure tacrolimus concentrations in peripheral blood CD4^+^ T cells and CD19^+^ B lymphocytes. Still, they did not evaluate the relationships between drug levels in these cell types and renal function or rejection. Similarly, Udomkarnjananun et al. (2022) studied kidney transplant recipients with acute rejection. They found no association between tacrolimus levels in CD3^+^ T lymphocytes or CD14^+^ monocytes and rejection, potentially due to small sample sizes and differences between fresh and frozen cells. These findings highlight the need for further research to determine how variations in tacrolimus levels across different PBMC cell types and whole blood influence clinical outcomes (Lemaitre et al., 2020; Udomkarnjananun et al., 2023). Additionally, factors such as CYP3A5/ABCB1 polymorphisms, ethnicity, and the type of organ transplantation may affect PBMC drug metabolism and response to tacrolimus (Wang et al., 2022; Tornatore et al., 2023).

Our results demonstrated that during the 1-year post-transplant period, as tacrolimus dosages progressively decreased, tacrolimus concentrations in PBMCs showed a continuous downward trend, distinct from whole-blood concentrations. PBMC tacrolimus levels were positively correlated with postoperative kidney function markers, including Ccr and eGFR. Notably, tacrolimus IPV in PBMCs was significantly higher in recipients with dnDSA than whole-blood IPV, suggesting that pharmacokinetic variations of tacrolimus in PBMCs may better reflect transplanted kidney function. This aligns with findings by Guo et al. (2024), who reported that monitoring intracellular tacrolimus concentrations in PBMCs is particularly critical in kidney transplant recipients with impaired renal function. Another study (van der Veer et al., 2019) discovered that a high IPV in tacrolimus exposure beyond 6 months after liver transplantation was not associated with immune-mediated graft injury. However, it was related to a decline in renal function in patients with impaired baseline renal function, suggesting that the IPV may vary across different patient populations. Specifically, elevated IPV is potentially associated with a decline in renal function among patients with pre-existing renal impairment. Since only two rejection patients were observe in our study, the association of IPV with allograft injury cannot be further evaluated.

Numerous studies have focused on elucidating the relationship between tacrolimus IPV and clinical outcomes. However, the interpretation of these findings remains confounded by methodological heterogeneity in IPV calculation (e.g., coefficient of variation vs dose-normalized metrics) and patient-specific factors, including genetic variations (e.g., CYP3A5 polymorphisms), comorbidities, and adherence patterns. To mitigate the impact of interindividual variability on IPV, our study normalized tacrolimus concentrations by using the daily tacrolimus dose and patient body weight. This approach accounts for differences in prescribed doses and body mass, thereby standardizing drug exposure metrics to more accurately reflect intrinsic metabolic capacity, which might be more valuable for evaluating the effect of tacrolimus IPV on clinical outcomes.

This study has several limitations. The relatively small sample size (n = 60), particularly in later follow-ups (e.g., Month 12, n = 17), may limit the statistical power of subgroup analyses, such as correlations between tacrolimus IPV and dnDSA/rejection. Despite the limitations of our study, the current evidence suggests that PBMC tacrolimus concentrations provide more clinically informative insights than whole-blood levels in certain contexts. To address the limitations of this study, future multi-center clinical investigations will be conducted to explore how different PBMC cell types respond to tacrolimus concentration variations. Additionally, advanced biomarkers for evaluating post-transplant immune status will be sought to optimize immunosuppressive regimens and improve kidney transplant recipients’ long-term outcomes and quality of life.

## 5 Conclusion

This prospective clinical study demonstrated, for the first time, that tacrolimus levels in PBMCs were positively correlated with those in whole blood, with PBMC tacrolimus levels showing a stronger association with transplanted kidney function. The study revealed that PBMC tacrolimus IPV in dnDSA-positive kidney transplant recipients was significantly higher than whole-blood tacrolimus IPV. A negative correlation between PBMC tacrolimus IPV and kidney function was identified for the first time. These findings address a critical gap in this field in China and provide a foundation for further research on the immunological mechanisms underlying ABMR in high-IPV and dnDSA-positive kidney transplant recipients.

## Data Availability

The raw data supporting the conclusions of this article will be made available by the authors, without undue reservation.

## Abbreviations

PBMC, peripheral blood mononuclear cell; IPV, intra-patient variability; dnDSA, *de novo* donor-specific antibody; ABMR, antibody-mediated rejection; TDM, therapeutic drug monitoring; C0, trough concentrations; Scr, serum creatinine; Ccr, creatinine clearance rate; eGFR, estimated glomerular filtration rate; CV, coefficient of variation; CMIA, chemiluminescent microparticle immunoassay; LC-MS/MS, liquid chromatography-tandem mass spectrometry; HLA, human leukocyte antigen; SD, standard deviation; GC, glucocorticoids; MPA, mycophenolic acid; TAC, Tacrolimus; TCMR, T cell-mediated rejection.
